# 4,4′-(Oxydimethyl­ene)dibenzonitrile

**DOI:** 10.1107/S1600536808020527

**Published:** 2008-07-09

**Authors:** Jie Xiao, Hong Zhao

**Affiliations:** a Ordered Matter Science Research Center, College of Chemistry and Chemical Engineering, Southeast University, Nanjing 210096, People’s Republic of China

## Abstract

The title compound, C_16_H_12_N_2_O, was accidentally synthesized by the reaction of 4-(bromo­meth­yl)benzonitrile and penta­erythritol. The dihedral angle between the benzene rings is 57.39 (9)°. In the crystal structure, mol­ecules are linked by inter­molecular C—H⋯N hydrogen-bonding inter­actions to form chains running parallel to the *b* axis.

## Related literature

For applications of nitrile derivatives in the synthesis of some heterocyclic mol­ecules, see: Radl *et al.* (2000 ▶); Jin *et al.* (1994 ▶). For the crystal structure of a related compound, see: Fu & Zhao (2007 ▶).
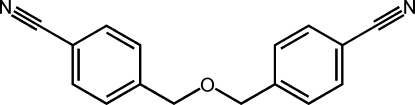


## Experimental

### 

#### Crystal data

C_16_H_12_N_2_O
*M* *_r_* = 248.28
Monoclinic, 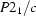

*a* = 14.444 (3) Å
*b* = 7.6674 (13) Å
*c* = 11.897 (2) Å
β = 96.326 (14)°
*V* = 1309.6 (4) Å^3^
*Z* = 4
Mo *K*α radiation
μ = 0.08 mm^−1^
*T* = 293 (2) K
0.35 × 0.30 × 0.30 mm

#### Data collection

Rigaku Mercury2 diffractometer
Absorption correction: multi-scan (*CrystalClear*; Rigaku, 2005 ▶) *T*_min_ = 0.939, *T*_max_ = 0.978
13064 measured reflections
3007 independent reflections
1498 reflections with *I* > 2σ(*I*)
*R*_int_ = 0.071

#### Refinement

*R*[*F* ^2^ > 2σ(*F* ^2^)] = 0.064
*wR*(*F* ^2^) = 0.149
*S* = 1.01
3007 reflections
172 parameters
H-atom parameters constrained
Δρ_max_ = 0.11 e Å^−3^
Δρ_min_ = −0.15 e Å^−3^

### 

Data collection: *CrystalClear* (Rigaku, 2005 ▶); cell refinement: *CrystalClear*; data reduction: *CrystalClear*; program(s) used to solve structure: *SHELXS97* (Sheldrick, 2008 ▶); program(s) used to refine structure: *SHELXL97* (Sheldrick, 2008 ▶); molecular graphics: *SHELXTL/PC* (Sheldrick, 2008 ▶); software used to prepare material for publication: *SHELXL97*.

## Supplementary Material

Crystal structure: contains datablocks I, global. DOI: 10.1107/S1600536808020527/rz2229sup1.cif

Structure factors: contains datablocks I. DOI: 10.1107/S1600536808020527/rz2229Isup2.hkl

Additional supplementary materials:  crystallographic information; 3D view; checkCIF report

## Figures and Tables

**Table 1:** Hydrogen-bond geometry (Å, °)

*D*—H⋯*A*	*D*—H	H⋯*A*	*D*⋯*A*	*D*—H⋯*A*
C14—H14⋯N2^i^	0.93	2.60	3.490 (3)	162

Symmetry code: (i) 
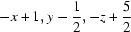
.

**Fig. 1.**
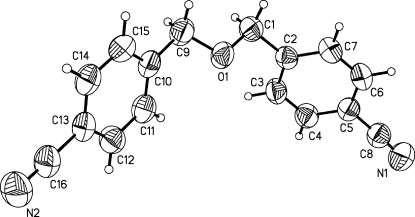
The molecular structure of the title compound, showing the atomic numbering scheme. Displacement ellipsoids are drawn at the 50% probability level.

## supplementary crystallographic information

### Comment

Nitrile derivatives are an important class of compounds used in the chemical industry. For example, nitrile derivatives are employed in the synthesis of some heterocyclic molecules (Radl *et al.*,  2000), and have been used as starting materials for the synthesis of phthalocyanines (Jin *et al.*, 1994). Recently, we have reported the crystal structure of a benzonitrile compound (Fu & Zhao, 2007). The title compound was unexpectedly obtained during our work on nitrile compounds, and its crystal structure is reported here.

In the title compound (Fig. 1), bond lengths and angles have normal values. The planes through the C2—C7 and C10—C15 benzene rings form a dihedral angle of 57.39 (9)°. The crystal structure is stabilized by an intermolecular C—H···N hydrogen bond forming chains of molecules along the b-axis (Table 1).

### Experimental

Pentaerythritol (0.136 g, 1 mmol) and 4-(bromomethyl)benzonitrile (0.658 g, 4 mmol) were dissolved in water in the presence of sodium hydroxide (0.160 g, 4 mmol) and heated under reflux for 2 days. After the mixture was cooled to room temperature, the solvent was removed in vacuum to afford a white precipitate of the title compound. Colourless crystals suitable for X-ray diffraction were obtained from a solution of 100 mg in 15 ml diethylether by slow evaporation after 5 days.

### Refinement

H atoms were positioned geometrically and refined using a riding model, with C—H = 0.93–0.97 Å and *U*_iso_(H) = 1.2*U*_eq_(C).

### Crystal data

**Table:**

C_16_H_12_N_2_O	*F*_000_ = 520
*M**_r_* = 248.28	*D*_x_ = 1.259 Mg m^−^^3^
Monoclinic, *P*2_1_/*c*	Mo *K*α radiation λ = 0.71073 Å
Hall symbol: -P 2ybc	Cell parameters from 1930 reflections
*a* = 14.444 (3) Å	θ = 2.8–27.5º
*b* = 7.6674 (13) Å	µ = 0.08 mm^−^^1^
*c* = 11.897 (2) Å	*T* = 293 (2) K
β = 96.326 (14)º	Block, colourless
*V* = 1309.6 (4) Å^3^	0.35 × 0.30 × 0.30 mm
*Z* = 4	

### Data collection

**Table:**

Rigaku Mercury2 diffractometer	3007 independent reflections
Radiation source: fine-focus sealed tube	1498 reflections with *I* > 2σ(*I*)
Monochromator: graphite	*R*_int_ = 0.072
Detector resolution: 13.6612 pixels mm^-1^	θ_max_ = 27.5º
*T* = 293(2) K	θ_min_ = 2.8º
ω scans	*h* = −18→18
Absorption correction: Multi-scan(CrystalClear; Rigaku, 2005)	*k* = −9→9
*T*_min_ = 0.939, *T*_max_ = 0.978	*l* = −15→15
13064 measured reflections	

### Refinement

**Table:**

Refinement on *F*^2^	Secondary atom site location: difference Fourier map
Least-squares matrix: full	Hydrogen site location: inferred from neighbouring sites
*R*[*F*^2^ > 2σ(*F*^2^)] = 0.064	H-atom parameters constrained
*wR*(*F*^2^) = 0.149	*w* = 1/[σ^2^(*F*_o_^2^) + (0.0573*P*)^2^] where *P* = (*F*_o_^2^ + 2*F*_c_^2^)/3
*S* = 1.01	(Δ/σ)_max_ < 0.001
3007 reflections	Δρ_max_ = 0.11 e Å^−^^3^
172 parameters	Δρ_min_ = −0.15 e Å^−^^3^
Primary atom site location: structure-invariant direct methods	Extinction correction: none

### Special details

**Table:**

Geometry. All e.s.d.'s (except the e.s.d. in the dihedral angle between two l.s. planes) are estimated using the full covariance matrix. The cell e.s.d.'s are taken into account individually in the estimation of e.s.d.'s in distances, angles and torsion angles; correlations between e.s.d.'s in cell parameters are only used when they are defined by crystal symmetry. An approximate (isotropic) treatment of cell e.s.d.'s is used for estimating e.s.d.'s involving l.s. planes.
Refinement. Refinement of *F*^2^ against ALL reflections. The weighted *R*-factor *wR* and goodness of fit *S* are based on *F*^2^, conventional *R*-factors *R* are based on *F*, with *F* set to zero for negative *F*^2^. The threshold expression of *F*^2^ > σ(*F*^2^) is used only for calculating *R*-factors(gt) *etc*. and is not relevant to the choice of reflections for refinement. *R*-factors based on *F*^2^ are statistically about twice as large as those based on *F*, and *R*- factors based on ALL data will be even larger.

### Fractional atomic coordinates and isotropic or equivalent isotropic displacement parameters (Å^2^)

**Table:**

	*x*	*y*	*z*	*U*_iso_*/*U*_eq_	
O1	0.21121 (10)	0.2174 (2)	0.82830 (14)	0.0632 (5)	
C13	0.40291 (15)	0.6678 (3)	1.0392 (2)	0.0543 (6)	
C5	−0.05516 (16)	0.2145 (3)	0.5362 (2)	0.0530 (6)	
C1	0.17453 (17)	0.0647 (3)	0.7737 (2)	0.0610 (7)	
H1A	0.1547	−0.0163	0.8289	0.073*	
H1B	0.2218	0.0082	0.7346	0.073*	
C16	0.43912 (17)	0.8289 (4)	1.0896 (2)	0.0647 (7)	
C3	0.10674 (17)	0.2247 (3)	0.6017 (2)	0.0616 (7)	
H3	0.1662	0.2664	0.5941	0.074*	
C2	0.09330 (16)	0.1156 (3)	0.69105 (19)	0.0505 (6)	
C11	0.33163 (17)	0.5062 (3)	0.8811 (2)	0.0608 (7)	
H11	0.3073	0.5020	0.8054	0.073*	
C7	0.00448 (17)	0.0575 (3)	0.7016 (2)	0.0592 (7)	
H7	−0.0057	−0.0156	0.7614	0.071*	
C4	0.03367 (17)	0.2726 (3)	0.5241 (2)	0.0624 (7)	
H4	0.0440	0.3439	0.4635	0.075*	
C10	0.33245 (15)	0.3570 (3)	0.9471 (2)	0.0539 (6)	
C6	−0.06975 (17)	0.1060 (3)	0.6249 (2)	0.0618 (7)	
H6	−0.1294	0.0657	0.6331	0.074*	
C9	0.29580 (16)	0.1870 (3)	0.8972 (2)	0.0659 (7)	
H9A	0.3410	0.1358	0.8524	0.079*	
H9B	0.2855	0.1062	0.9572	0.079*	
C12	0.36687 (17)	0.6610 (3)	0.9274 (2)	0.0625 (7)	
H12	0.3662	0.7608	0.8828	0.075*	
C14	0.40499 (17)	0.5201 (3)	1.1048 (2)	0.0619 (7)	
H14	0.4302	0.5241	1.1801	0.074*	
C15	0.36940 (16)	0.3653 (3)	1.0584 (2)	0.0625 (7)	
H15	0.3705	0.2657	1.1031	0.075*	
C8	−0.13138 (19)	0.2686 (3)	0.4553 (2)	0.0640 (7)	
N2	0.46806 (17)	0.9545 (3)	1.1316 (2)	0.0850 (8)	
N1	−0.19108 (17)	0.3162 (3)	0.3912 (2)	0.0883 (8)	

### Atomic displacement parameters (Å^2^)

**Table:**

	*U*^11^	*U*^22^	*U*^33^	*U*^12^	*U*^13^	*U*^23^
O1	0.0528 (10)	0.0591 (10)	0.0733 (11)	0.0012 (8)	−0.0121 (8)	−0.0152 (8)
C13	0.0438 (13)	0.0670 (16)	0.0511 (15)	−0.0015 (11)	0.0004 (11)	−0.0064 (12)
C5	0.0529 (15)	0.0533 (14)	0.0521 (15)	0.0004 (11)	0.0024 (12)	−0.0107 (11)
C1	0.0591 (16)	0.0546 (15)	0.0677 (17)	−0.0037 (12)	0.0000 (13)	−0.0063 (12)
C16	0.0568 (16)	0.0752 (19)	0.0604 (17)	−0.0035 (13)	−0.0009 (13)	−0.0087 (14)
C3	0.0477 (15)	0.0689 (17)	0.0690 (17)	−0.0086 (12)	0.0107 (13)	0.0035 (13)
C2	0.0524 (14)	0.0463 (13)	0.0527 (15)	−0.0039 (11)	0.0050 (11)	−0.0096 (11)
C11	0.0572 (16)	0.0742 (18)	0.0479 (15)	−0.0013 (12)	−0.0078 (12)	−0.0039 (13)
C7	0.0594 (16)	0.0601 (16)	0.0586 (16)	−0.0125 (12)	0.0082 (13)	−0.0004 (12)
C4	0.0607 (17)	0.0676 (17)	0.0600 (16)	−0.0025 (13)	0.0117 (13)	0.0057 (12)
C10	0.0405 (13)	0.0632 (16)	0.0567 (15)	0.0052 (11)	−0.0002 (11)	−0.0076 (12)
C6	0.0525 (15)	0.0656 (17)	0.0678 (17)	−0.0138 (12)	0.0086 (13)	−0.0060 (13)
C9	0.0538 (15)	0.0664 (17)	0.0743 (18)	0.0051 (12)	−0.0065 (13)	−0.0083 (13)
C12	0.0646 (16)	0.0652 (17)	0.0558 (16)	−0.0057 (13)	−0.0020 (13)	0.0029 (12)
C14	0.0548 (16)	0.0772 (19)	0.0508 (15)	0.0023 (13)	−0.0072 (12)	−0.0038 (13)
C15	0.0580 (15)	0.0650 (17)	0.0619 (17)	0.0042 (12)	−0.0044 (13)	0.0063 (13)
C8	0.0621 (17)	0.0638 (17)	0.0654 (18)	−0.0015 (13)	0.0041 (14)	−0.0083 (13)
N2	0.0928 (19)	0.0813 (18)	0.0772 (17)	−0.0108 (14)	−0.0071 (14)	−0.0135 (14)
N1	0.0732 (16)	0.0958 (18)	0.0919 (19)	0.0035 (14)	−0.0094 (14)	0.0044 (15)

### Geometric parameters (Å, °)

**Table:**

O1—C9	1.414 (3)	C11—C12	1.382 (3)
O1—C1	1.414 (2)	C11—C10	1.387 (3)
C13—C14	1.374 (3)	C11—H11	0.9300
C13—C12	1.375 (3)	C7—C6	1.380 (3)
C13—C16	1.445 (3)	C7—H7	0.9300
C5—C6	1.379 (3)	C4—H4	0.9300
C5—C4	1.380 (3)	C10—C15	1.373 (3)
C5—C8	1.441 (3)	C10—C9	1.504 (3)
C1—C2	1.497 (3)	C6—H6	0.9300
C1—H1A	0.9700	C9—H9A	0.9700
C1—H1B	0.9700	C9—H9B	0.9700
C16—N2	1.142 (3)	C12—H12	0.9300
C3—C4	1.374 (3)	C14—C15	1.384 (3)
C3—C2	1.384 (3)	C14—H14	0.9300
C3—H3	0.9300	C15—H15	0.9300
C2—C7	1.377 (3)	C8—N1	1.145 (3)
			
C9—O1—C1	112.70 (17)	C3—C4—C5	119.9 (2)
C14—C13—C12	120.0 (2)	C3—C4—H4	120.1
C14—C13—C16	119.0 (2)	C5—C4—H4	120.1
C12—C13—C16	121.0 (2)	C15—C10—C11	119.1 (2)
C6—C5—C4	119.8 (2)	C15—C10—C9	120.2 (2)
C6—C5—C8	121.0 (2)	C11—C10—C9	120.7 (2)
C4—C5—C8	119.2 (2)	C5—C6—C7	119.7 (2)
O1—C1—C2	108.22 (18)	C5—C6—H6	120.1
O1—C1—H1A	110.1	C7—C6—H6	120.1
C2—C1—H1A	110.1	O1—C9—C10	109.29 (19)
O1—C1—H1B	110.1	O1—C9—H9A	109.8
C2—C1—H1B	110.1	C10—C9—H9A	109.8
H1A—C1—H1B	108.4	O1—C9—H9B	109.8
N2—C16—C13	178.5 (3)	C10—C9—H9B	109.8
C4—C3—C2	121.0 (2)	H9A—C9—H9B	108.3
C4—C3—H3	119.5	C13—C12—C11	120.1 (2)
C2—C3—H3	119.5	C13—C12—H12	119.9
C7—C2—C3	118.5 (2)	C11—C12—H12	119.9
C7—C2—C1	121.8 (2)	C13—C14—C15	119.8 (2)
C3—C2—C1	119.7 (2)	C13—C14—H14	120.1
C12—C11—C10	120.2 (2)	C15—C14—H14	120.1
C12—C11—H11	119.9	C10—C15—C14	120.8 (2)
C10—C11—H11	119.9	C10—C15—H15	119.6
C2—C7—C6	121.1 (2)	C14—C15—H15	119.6
C2—C7—H7	119.4	N1—C8—C5	178.1 (3)
C6—C7—H7	119.4		

### Hydrogen-bond geometry (Å, °)

**Table:**

*D*—H···*A*	*D*—H	H···*A*	*D*···*A*	*D*—H···*A*
C14—H14···N2^i^	0.93	2.60	3.490 (3)	162

Symmetry codes: (i) −*x*+1, *y*−1/2, −*z*+5/2.
